# Regulation of inflammatory responses by neuregulin-1 in brain ischemia and microglial cells in vitro involves the NF-kappa B pathway

**DOI:** 10.1186/s12974-016-0703-7

**Published:** 2016-09-06

**Authors:** Lauren J. Simmons, Monique C. Surles-Zeigler, Yonggang Li, Gregory D. Ford, Gale D. Newman, Byron D. Ford

**Affiliations:** 1 Morehouse School of Medicine, 720 Westview Drive, Atlanta, 30310 Georgia; 2 Division of Biomedical Sciences, University of California—Riverside School of Medicine, 900 University Ave, Riverside, CA 92521 USA; 3 Georgia Highlands College, 5441 GA-20, Cartersville, GA 30121 USA

**Keywords:** Bioinformatics, Gene expression, Inflammation, Ischemia, Microarray, Neuregulin, Stroke, Transcription factor-binding site (TFBS)

## Abstract

**Background:**

We previously demonstrated that neuregulin-1 (NRG-1) was neuroprotective in rats following ischemic stroke. Neuroprotection by NRG-1 was associated with the suppression of pro-inflammatory gene expression in brain tissues. Over-activation of brain microglia can induce pro-inflammatory gene expression by activation of transcriptional regulators following stroke. Here, we examined how NRG-1 transcriptionally regulates inflammatory gene expression by computational bioinformatics and in vitro using microglial cells.

**Methods:**

To identify transcriptional regulators involved in ischemia-induced inflammatory gene expression, rats were sacrificed 24 h after middle cerebral artery occlusion (MCAO) and NRG-1 treatment. Gene expression profiles of brain tissues following ischemia and NRG-1 treatment were examined by microarray technology. The Conserved Transcription Factor-Binding Site Finder (CONFAC) bioinformatics software package was used to predict transcription factors associated with inflammatory genes induced following stroke and suppressed by NRG-1 treatment. NF-kappa B (NF-kB) was identified as a potential transcriptional regulator of NRG-1-suppressed genes following ischemia. The involvement of specific NF-kB subunits in NRG-1-mediated inflammatory responses was examined using N9 microglial cells pre-treated with NRG-1 (100 ng/ml) followed by lipopolysaccharide (LPS; 10 μg/ml) stimulation. The effects of NRG-1 on cytokine production were investigated using Luminex technology. The levels of the p65, p52, and RelB subunits of NF-kB and IkB-α were determined by western blot analysis and ELISA. Phosphorylation of IkB-α was investigated by ELISA.

**Results:**

CONFAC identified 12 statistically over-represented transcription factor-binding sites (TFBS) in our dataset, including NF-kBP65. Using N9 microglial cells, we observed that NRG-1 significantly inhibited LPS-induced TNFα and IL-6 release. LPS increased the phosphorylation and degradation of IkB-α which was blocked by NRG-1. NRG-1 also prevented the nuclear translocation of the NF-kB p65 subunit following LPS administration. However, NRG-1 increased production of the neuroprotective cytokine granulocyte colony-stimulating factor (G-CSF) and the nuclear translocation of the NF-kB p52 subunit, which is associated with the induction of anti-apoptotic and suppression of pro-inflammatory gene expression.

**Conclusions:**

Neuroprotective and anti-inflammatory effects of NRG-1 are associated with the differential regulation of NF-kB signaling pathways in microglia. Taken together, these findings suggest that NRG-1 may be a potential therapeutic treatment for treating stroke and other neuroinflammatory disorders.

## Background

The actions of neuregulins have been demonstrated in normal brain function and in neuroprotection following cerebral ischemia. Studies from our laboratory and others demonstrated that neuregulin-1 (NRG-1) reduced ischemia-induced neuronal death in rodent models of focal stroke [1–3]. NRG-1 was neuroprotective with a therapeutic window of >13 h and significantly improved neurological function [2, 4]. Neuroprotection by NRG-1 was also associated with the inhibition of pro-inflammatory gene expression following ischemia [2, 3, 5–8]. The anti-inflammatory effects of NRG-1 have also been shown in other animal models of neuroinflammation including cerebral malaria and nerve agent intoxication [9, 10] and in vivo using cultured macrophages and microglia [3, 11]. However, the cellular and molecular mechanisms involved in the immunomodulatory effects of NRG-1 remain unclear.

Neuronal death following ischemic stroke involves the induction of genes associated with a number of cellular functions, including apoptosis, inflammation, and oxidative stress [12, 13]. The initial inflammatory response following brain injury is mediated by the activation of its resident inflammatory cells, the microglia [14–16]. Activated microglia release inflammatory mediators such as reactive oxygen species (ROS), nitric oxide (NO), tumor necrosis factor α (TNF-α), and interleukin-1β (IL-1β) [17–19]**.** While the initial activation of microglia is the brain’s attempt to protect neurons, the over-activation of microglia leads to uncontrolled inflammation which exacerbates neuronal death [18, 20, 21]. Nuclear factor kappa B (NF-kB) is a transcription factor widely known to be associated with inflammatory responses following ischemia and other neuroinflammatory disorders [22–24]. There are five members of the NF-kB family which include p65 (RelA), RelB, c-Rel, p50/105 (NF-kB1), and p52/p100 (NF-kB2), which exist in unstimulated cells as homo or heterodimers bound to inhibitory kB (IkB) family proteins**.** Activation of the canonical NF-kB pathway triggers IkB kinase (IKK) activity, leading to phosphorylation and degradation of IkB proteins and the release of NF-kB p65/p50 heterodimers. The released p65/p50 NF-kB dimers translocate from the cytoplasm to the nucleus where they bind to specific DNA sequences and promote transcription of target genes [23, 24]. In the canonical pathway, NF-kB activation triggers the production of pro-inflammatory cytokines and NO [18, 25, 26]. Studies have shown that inhibition of the canonical NF-kB pathway prevents the production of pro-inflammatory cytokines [27–29]. The activation of the NF-kB alternative/non-canonical pathway involves NF-kB-inducing kinase (NIK), a member of the MAP kinase family, which activates IKKα, resulting in the translocation of RelB/p52 NF-kB heterodimers to the nucleus [30–34]. The alternative NF-kB pathway has been shown to stimulate the production of anti-apoptotic and anti-inflammatory molecules [31, 35–39]. The effects of NRG-1 have been shown to be mediated by NF-kB signaling during tumorigenesis in cancers and axonal myelination [40–45]. Specifically, NRG-1 has been shown to activate NIK through its association with the NRG-1’s erbB receptors [46].

To identify transcriptional regulators involved in ischemia-induced inflammatory gene expression, we examined gene expression profiles of brain tissues following ischemia and NRG-1 treatment. Using Conserved Transcription Factor-Binding Site Finder (CONFAC) software, we performed computational analysis to predict transcriptional regulators of genes that were induced following ischemic stroke but were downregulated by NRG-1. CONFAC identifies conserved transcription factor-binding sites significantly over-represented in promoter regions of a set of genes of interest compared to random control set of genes [47]. NF-kB was identified as one of the top potential regulators of NRG-1-suppressed genes following ischemia. Using N9 microglial cells, we observed that NRG-1 blocked the phosphorylation and degradation of IkB-α, leading to the attenuation of classical NF-kB activation. NRG-1 increased the nuclear translocation of the p52 subunit of NF-kB and the levels of anti-apoptotic cytokines G-CSF and IL-9, suggesting a role for the alternative NF-kB pathway. Understanding these mechanisms will provide insight into the molecular processes behind the neuroprotective and anti-inflammatory abilities of NRG-1.

## Methods

### Transient middle cerebral artery occlusion (MCAO)

All animals were treated humanely and with regard for alleviation of suffering and pain. All surgical protocols involving animals were performed by sterile/aseptic techniques and were approved by the Institutional Animal Care and Use Committee at Morehouse School of Medicine prior to the initiation of experimentation. Adult male Sprague-Dawley rats (250–300 g; Charles River Laboratory International, Inc., USA) were housed in standard cages in a temperature-controlled room (22 ± 2 °C) on a 12-h reverse light-dark cycle. Food and water were provided ad libitum.

Animals were randomly allocated into three groups: sham (control), middle cerebral artery occlusion (MCAO) + vehicle treatment (MCAO) and MCAO + NRG-1 (MCAO + NRG1). Rats were anesthetized with a ketamine/xylazine solution (100/10 mg/kg, i.p.) prior to surgery. After anesthesia administration, a rectal probe monitored the core body temperature and a Homoeothermic Blanket Control Unit (Harvard Apparatus, Hollister, MA) was used to ensure the body temperature maintained at 37 °C. Cerebral blood flow was monitored throughout the length of the surgery by a continuous laser Doppler flowmeter (Perimed, Ardmore, PA), with a laser Doppler probe placed 7 mm lateral and 2 mm posterior to bregma in a thinned cranial skull window.

Rats in the treatment groups (MCAO and MCAO + NRG1) were subjected to a left transient MCAO. MCAO was induced by the intraluminal suture method as previously described [3]. Briefly, a 4-cm length 4-0 surgical monofilament nylon suture coated with silicon (Doccol Corp., Sharon, MA) was inserted from the external carotid artery (ECA) into the internal carotid artery (ICA) and then into the Circle of Willis, to occlude the origin of the left middle cerebral artery (MCA). After 1.5 h of ischemia, the nylon suture was removed and the ischemic brain was reperfused for 22.5 h before sacrifice. Rats in the sham control group underwent the same procedure as those in the treatment groups, but a filament was not inserted into the ICA. Animals were randomly assigned into treatment groups and either administered 50 μl of NRG-1β reconstituted with 1 % bovine serum albumin (BSA) in phosphate-buffered saline (PBS) (MCAO + NRG1; 20 μg/kg; EGF-like domain, R&D Systems, Minneapolis, MN) or vehicle (MCAO; 1 % BSA in PBS) All treatments were administered by bolus injection into the ICA through ECA immediately before MCAO. Animals were sacrificed 24 h after MCAO. All NRG-1 and vehicle treatment studies were performed in a blinded manner.

### RNA preparation and microarray analysis

Microarray analysis was performed as we previously described [3, 48, 49]. Animals were sacrificed, and their brains removed 24 h following MCAO. Removed brains were sliced into 2-mm coronal sections (approximately +3.0 to −5.0 from bregma) using a brain matrix. Tissue from the ipsilateral tissue from the two middle slices (+1 to −3 from bregma) of MCAO treated (*n* = 3) and MCAO + NRG1 treated (*n* = 3) and sham animals (*n* = 3) were used for RNA isolation, while the two outer slices of tissue were used for staining with 2,3,5-triphenyltetrazolium chloride (TTC), in order to confirm infarct formation. Total RNA extraction was performed using TRIzol Reagent (Life Technologies, Rockville, MD, USA), cleaned (RNAqueous Kit Ambion, Austin, TX, USA), and converted to double-stranded complementary DNA (cDNA) (Invitrogen, Superscript Choice System, Carlsbad, CA, USA) using T7-(dT)24 primer. Cleanup of double-stranded cDNA used Phase Lock Gels (Eppendorf, Westbury, NY, USA)-Phenol/Chloroform/Isoamyl Alcohol (Sigma, St. Louis, MO, USA). cRNA was synthesized using a RNA transcript labeling kit (Enzo Diagnostics, Farmingdale, NY, USA). Biotin-labeled cRNA was cleaned up using a GeneChip Sample Cleanup Module (Affymetrix Inc., Santa Clara, CA, USA) and quantified using a spectrophotometer. Twenty micrograms of the in vitro transcription product was fragmented by placing at 94 °C for 35 min in fragmentation buffer. Following fragmentation, 15 μg of the biotinylated cRNA was hybridized to an Affymetrix Rat Genome U34A GeneChip. The chips were hybridized at 45 °C for 16 h, and then washed, stained with streptavidin-phycoerythrin, and scanned according to the manufacturing guidelines.

### Microarray data analysis

Affymetrix Expression Console software (Affymetrix, Santa Clara, CA) was used for initial data processing. Affymetrix microarrays contain the hybridization, labeling, and housekeeping controls that help determine the success of the hybridizations. The Affymetrix Expression Analysis algorithm uses the Tukey’s biweight estimator to provide a robust mean signal value and the Wilcoxon’s rank test to calculate a significance or *p* value and detection call for each probe set. The detection *p* value is calculated using a discrimination score (R) for all probes. The discrimination score is a basic property of a probe pair that describes its ability to detect its intended target. It measures the target-specific intensity differences of the probe pair (perfect match (PM)–mismatch (MM)) relative to its overall hybridization intensity (PM + MM). Background estimation is provided by a weighted average of the lowest 2 % of the feature intensities. Mismatch probes are utilized to adjust the PM intensity. Linear scaling of the feature level intensity values, using the trimmed mean, is the default to make the means equal for all arrays being analyzed. False-negative and false-positive rates are minimized by subtracting nonspecific signal from the PM probe intensities and performing an intensity-dependent normalization at the probe set level. Calculation of fold change and the presence of genes in the tissue were analyzed in Microsoft Excel. Changes in gene expression were compared between the control (sham) and following MCAO and MCAO + NRG-1 treatment. Gene expression values that increased or decreased by twofold or more were statistically significant (*p* < 0.05) using two-way ANOVA. The list of genes upregulated twofold or more by ischemia and decreased 50 % or more by NRG-1 was analyzed using Ingenuity Pathway Analysis (IPA) software (Qiagen, Redwood City, CA; www.ingenuity.com) overlaid onto a global molecular network developed from information contained in the Ingenuity Knowledge Base. Fischer’ s exact test was used to calculate a *p* value determining the probability that each biological function and/or disease assigned to that network is due to chance alone. The functions, canonical pathways, and gene networks that were most significant to the dataset were identified. IPA identified 64 inflammatory genes that were upregulated twofold or more by MCAO and reduced 50 % or more by NRG-1.

### CONFAC analysis

Using CONFAC software, we further examined the IPA-identified inflammatory genes [47, 48]. A tab-delimited text file containing the gene name and RefSEQ ID of the clusters of interest (genes) was uploaded to the CONFAC web browser interface (http://confac.emory.edu/). CONFAC software identified mouse orthologs from the uploaded gene list (from UCSC and ENSEMBL genomes). CONFAC software next identified significantly conserved sequences (*e* value <0.001), which were analyzed for transcription factor-binding sites (TFBS) using MATCH™ software. The final output table consisted of the cohort list of genes (column) and the position weight matrix (collected in TRANSFAC® database) identified potential TFBS and the number of TFBS determined for each gene of interest. The CONFAC program then allowed for additional analysis using the Mann-Whitney *U* test, which facilitated the statistical analysis of TFBS over-represented in the sample dataset compared to the seven control random control datasets provided by CONFAC.

### N9 microglial cell culture

The murine N9 microglial cell line was kindly provided by Dr. Celine Beamer (Department of Biomedical and Pharmaceutical Sciences, University Montana). N9 cells were cultured in Dulbecco’s modified Eagle’s media (DMEM) with 4 mM l-glutamine, supplemented with 10 % fetal bovine serum (FBS), streptomycin (100 U/ml), and penicillin (100 U/ml). The cells were maintained in 95 % air and 5 % CO_2_-humified atmosphere at 37 °C and were passaged every 3 days. For culturing with various stimulants, N9 cells were plated into 12-well plates in prepared culture medium and NRG-1 and lipopolysaccharide (LPS) were applied at the following final concentrations: LPS (10 μg/ml) and NRG-1 (100 ng/ml in 1 % BSA/PBS; recombinant human neuregulin1-beta1 EGF-like domain, R&D Systems). N9 cells were either pre-treated with NRG-1 (100 ng/ml) for 24 h followed by stimulation with LPS (10 μg/ml) or treated with LPS or NRG-1 alone for the indicated times (1–24 h).

### Luminex multiplex enzyme-linked immunosorbent assay (ELISA)

Cell lysates were taken at 1, 3, 6, and 24 h time points following LPS treatment. The cytokine release was measured in cell culture supernatants by Luminex technology using the manufacturer’s protocol (Bio-Rad, Hercules, CA). Data analysis was performed using Bio-Plex Manager software on the Bio-Plex 200 system. Experiments were performed four times in duplicate wells.

### Protein isolation and western blot

Proteins were isolated using whole cell extraction reagent M-PER (Pierce Biotechnology, Rockford, IL) or NE-PER Nuclear and Cytoplasmic Extraction Reagent (Thermo Scientific) according to the manufacturer’s instructions. Protein concentrations were determined by Bradford protein assay (Bio-Rad). Fifty micrograms of protein lysate was subjected to sodium dodecyl sulfate-polyacrylamide gel electrophoresis (SDS-PAGE) and transferred to polyvinylidene difluoride membranes (PVDF) (Bio-Rad, CA). The membranes were blocked with 3 % nonfat dry milk in 0.1 % Tween-20/Tris-buffered saline for 1 h at room temperature and subsequently incubated with the primary antibodies overnight, followed by incubation in corresponding secondary horseradish peroxidase-conjugated secondary antibody for 1 h at room temperature. Blots were developed using immuno-western star ECL detection kits (Bio-Rad). The optical densities of the antibody-specific bands were analyzed by a Luminescent Image Analyzer, LAS-4000 (Fuji, Japan). The following antibodies from Santa Cruz Biotechnology (Santa Cruz, CA, USA) were used: NF-kB p65 (sc-372; 1:200), IkB-α (sc-371; 1:200), YY1 (sc-281; 1:200), and β-actin (sc-130656; 1:200).

### IkB-α (total/phospho) InstantOne ELISA

The phosphorylation of IkB-α was determined using an enzyme-linked immunosorbent assay (ELISA)-based assay kit according to the manufacturer’s protocol (Affymetrix eBioscience, San Diego, CA). To analyze the results, we calculated the average values for each treatment group. This assay was run four times in duplicate.

### Active motif transAM NF-kB family ELISA

Nuclear extracts were isolated using NE-PER Nuclear and Cytoplasmic Extraction kit according to the manufacturer’s protocol (Thermo Scientific, Waltham, MA). ELISA was performed using the manufacturer’s protocol. To analyze the results, the optical density was read using 450 nm with an optional reference wavelength of 655 nm. This assay was run three times in duplicate.

### Statistical analysis

Statistical analysis for ELISAs and western blot data was performed by ANOVA with Tukey’s post hoc test for multiple comparisons where indicated. Significance was determined using a *p* value less than 0.05.

## Results

### CONFAC analysis predicts transcription factor NF-kB to be regulated following MCAO + NRG1

**Fig. 1:**
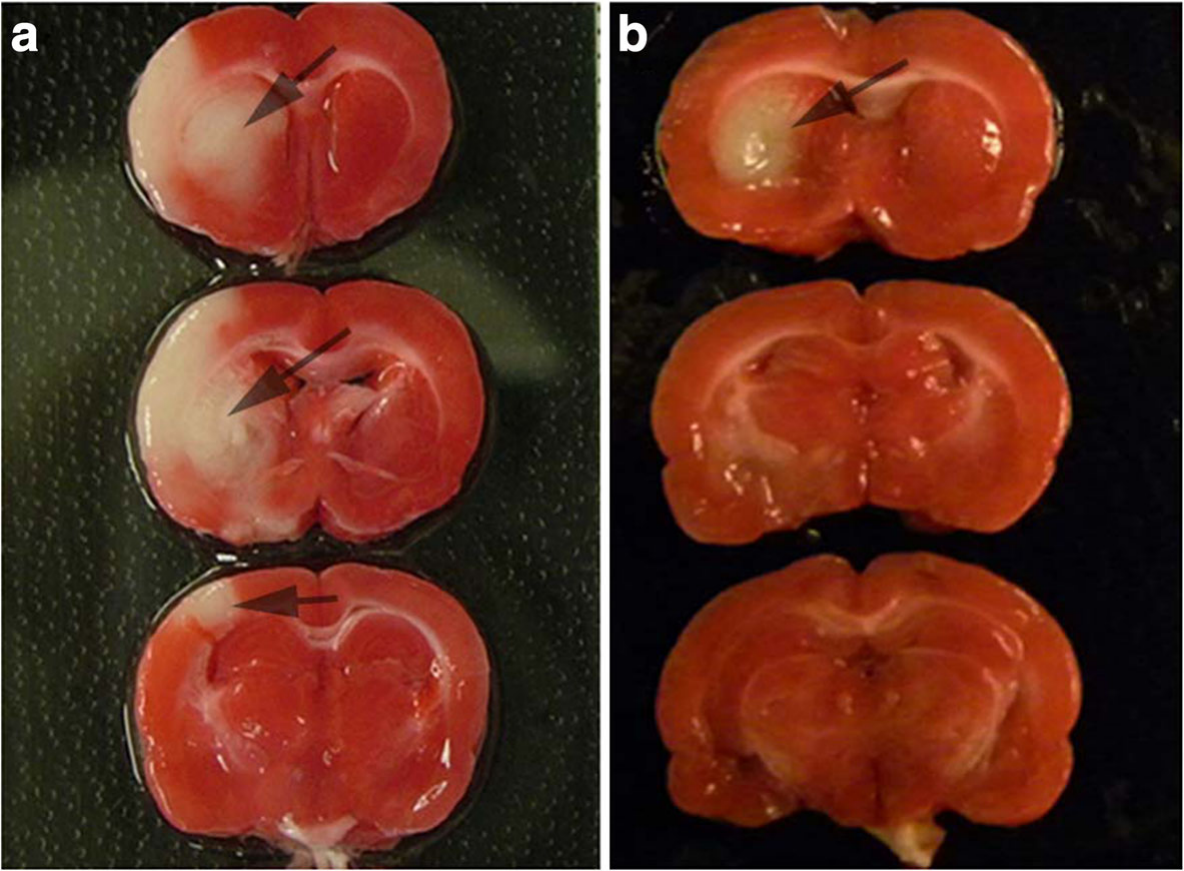
Neuregulin-1 administration reduces MCAO/reperfusion-induced brain infarction. Representative TTC-stained coronal brain sections are shown. Rats were administered vehicle (**a**) or NRG-1 before MCAO (**b**). The *white area* indicates damaged neuronal cells (*arrows*) and *red staining* indicates normally functioning cells

**Fig. 2:**
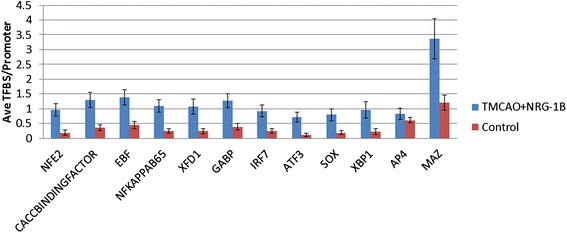
Predicted transcription factor-binding site (TFBS) activity for gene promoters using CONFAC analysis. CONFAC compared our gene list to seven random control datasets to identify statistically over-represented TFBS in genes altered by stroke and reversed by NRG-1. CONFAC identified 12 TFBS that were statistically over-represented. *Blue bars* represent the average number of TFBS/promoter for each transcription factor in our data set. *Red bars* are the average number of TFBS/promoter for each transcription factor in the control datasets. *p* < 0.05 for all transcription factors in the graph

NRG-1 has been shown to prevent neuronal injury when administered before or after MCAO. In these studies, rats were treated with NRG-1 immediately prior to transient MCAO for maximal effect. In representative TTC-stained coronal brain sections, rats were administered vehicle (Fig. 1a) or NRG-1 before MCAO (Fig. 1b). The white area indicates damaged neuronal cells (arrows) and red staining indicates normally functioning cells. RNA was isolated from the ipsilateral hemisphere of each experimental group (sham controls, MCAO, and MCAO + NRG1) and used to examine gene expression profiles in brain tissues from each condition. Using microarray and IPA analysis, we found that 64 inflammatory genes were increased twofold or more 24 h following MCAO and reduced by 50 % with NRG-1 treatment (Table 1). The list included a number of well-characterized pro-inflammatory molecules, including IL-1β, PTGS2/COX2 (protaglandin-endoperoxide synthase-2/cyclooxygenase-2), CD36, SPP1 (secreted phosphoprotein 1/osteopontin), and S100 calcium-binding proteins. IL-1β and PTGS2/COX2 induction by MCAO and in a monocytic cell line, respectively, and attenuation by NRG-1 were previously validated by qPCR [3]. CONFAC analysis software recognized 54 genes in our dataset and analyzed TFBS in the promoters of those genes. CONFAC identified 12 TFBS as over-represented in our dataset compared to the random control datasets. The TFBS included MAZ, NFKAPPAB65, XBP1, XFD1, SOX, ATF3, NFE2, IRF7, GABP, EBF, and CACCBINDINGFACTOR (Fig. 2). NFKAPPAB65 (NF-kB p65 subunit) has been previously associated with stroke and the effects of NRG-1 in breast cancer and neuronal myelination [40–43, 45], so we further investigated the role of NF-kB in NRG-1-mediated neuroinflammation.

**Table 1:** Inflammatory genes induced by ischemia and downregulated by NRG-1

A kinase anchor protein 12	Akap12
Actin-related protein 2/3 complex subunit 1B	Arpc1b
Activating transcription factor 3	Atf3
Annexin A1	Anxa1
Annexin A2	Anxa2
Benzodiazepine receptor (peripheral)-associated protein 1	bzrp
Brain-derived factor	Bdnf
CAMP responsive element modulator	Crem
Cartilage oligomeric matrix protein	Comp
Catechol-*O*-methyltransferase	Comt
CCAAT/enhancer-binding protein, delta	Cebpd
CD36 antigen	cd36
Cellular retinoic-binding protein 2	Crabp2
Crystallin, alpha B	Cryab
Cytochrome P450 family 1	Cyp1b1
Deiodinase iodothyronine type III	Dio3
Dyskeratosis congenita 1	Dkc1
Early growth response 1	Egr1
Endothelin converting enzyme 1	Ece1
Fx receptor, IgG, low-affinity III	Fcgr3
Galanin	Gal
Glycosylation-dependent cell adhesion molecule 1	Glycam1
Guanine nucleotide-binding protein gamma 11	Gng11
Guanylate-binding protein	Gbp2
Heat shock protein 4	Hspa4
High mobility group of box 2	Hmgb2
Homer scaffolding protein 1	Homer1
Inhibitor of DNA binding 1	Id1
Insulin-like growth factor-binding protein 3	Igfbp3
Interleukin 1 beta	IL1b
Isopentenyl-diphosphate delta isomerase 1	Idi1
Kininogen 1	Kng
Kruppel-like factor 4	Klf4
Lectin galatoside-binding soluble 2	LgalS2
Lipocalin 2	Lcn
Lipopolysaccharide-binding protein	Lbp
Lysozyme	Lyz
Matrix gla protein	Mgp
Matrix metallopeptidase 9	MMP9
Mitogen-activated protein kinase e, E3	Map
Oxidized low-density lipoprotein receptor 1	Olr1
Phospholipase A1	Pspla1
Phosphoribosyl pyrophosphate synthase-associated protein 1	Prpsap1
Phosphorylase glycogen liver	Pygl
Plasminogen activator tissue	Plat
Potassium channel member 12	Kcnj12
Potassium channel, two pore domain subfamily K member 3	Kcnk3
Protaglandin-endoperoxide synthase 2	Ptgs2
Rentinol-binding protein 1	Rbp1
Ret proto-oncogene	Ret
Ribosomal protein S15	Rps15
S100 calcium-binding protein A10	S100a10
S100 calcium-binding protein A4	S100a4
S100 calcium-binding protein A8	S100a8
S100 calcium-binding protein A9	S100a9
Secreted phosphoprotein 1	Spp1
Serpin peptidase inhibitor	Serpin1
Syndecan 1	Sdc1
Thyrotropin releasing hormone	Trh
TIMP metallopeptidase inhibitor 1	Timp1
Transgelin	Tagln
V-ETS avian erythroblastosis virus E26 oncogene homolog 1	ETS1
VGF nerve growth factor inducible	Vgf
Vimentin	Vim

#### NRG-1 suppressed the release of cytokines TNF-α and IL-6 in LPS-stimulated N9 microglial cells

**Fig. 3:**
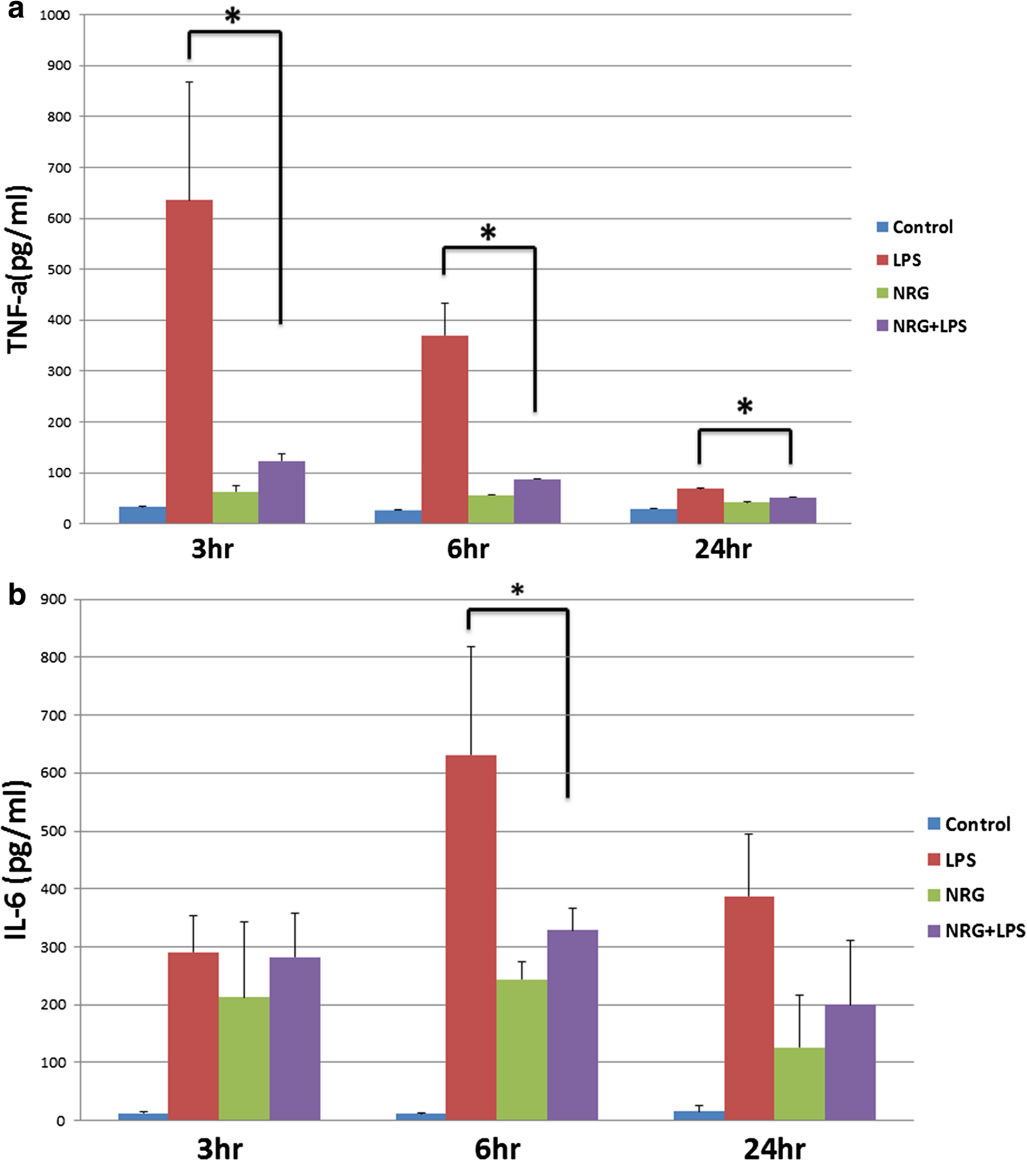
NRG-1 suppresses TNF-α and IL-6 concentrations in LPS-stimulated N9 microglia cells. N9 microglia cells were pre-treated with NRG-1 (100 ng/ml) for 24 h with or without LPS stimulation (10 μg/ml) for the indicated time points. Supernatants were collected, and TNF-α (**a**) and IL-6 (**b**) levels were determined by Luminex. Results are expressed as the mean +/− SD. *Asterisk* denotes a significant difference compared to cells treated with only LPS (*p* < 0.05)

We previously demonstrated the induction of IL-1β and monocyte chemoattractant protein 1 (MCP-1/CCL2) following MCAO and their inhibition by NRG-1 [3]. To determine whether NRG-1 inhibits the general production and release of pro-inflammatory cytokines, N9 microglia were pre-treated with NRG-1 followed by stimulation with LPS. Cytokine levels in the conditioned medium were measured by Luminex multiplex ELISA. Following LPS stimulation, there was an increase in TNF-α production and release at 3, 6, and 24 h from microglial cells. NRG-1 pre-treatment significantly reduced the levels of TNF-α at 3 h (*p* = 0.0003), 6 h (*p* = 0.0011), and 24 h (*p* = 0.01) post-stimulation with LPS (Fig. 3a). LPS stimulation also increased IL-6, which was significantly reduced by NRG-1 at 6 h (*p* = 0.02) post-LPS stimulation (Fig. 3b).

#### NRG-1 attenuated LPS-stimulated phosphorylation and degradation of IkB-α in N9 microglial cells

**Fig. 4:**
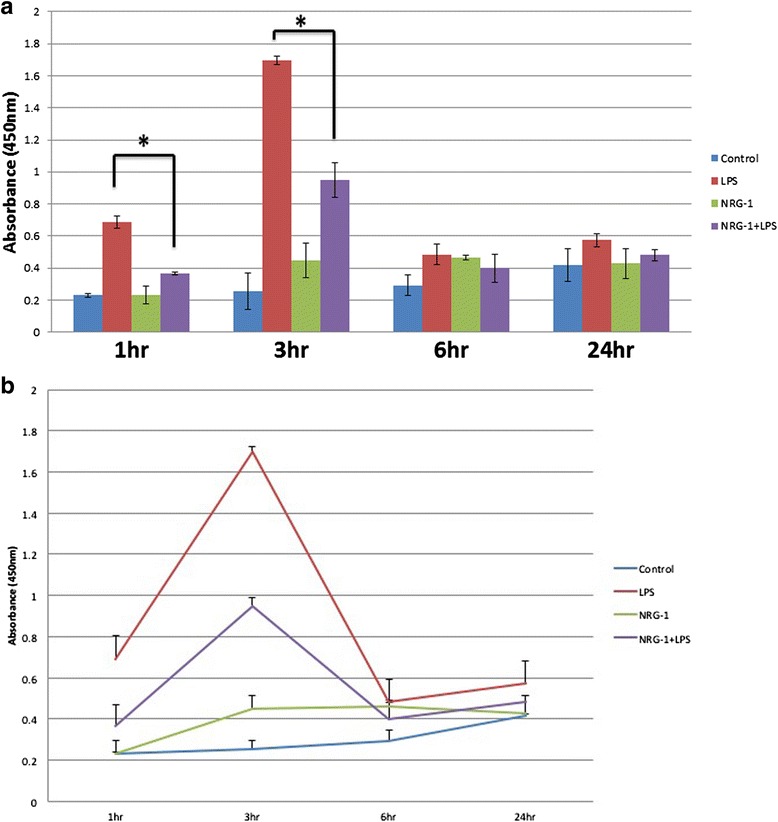
NRG-1 suppresses the phosphorylation of IkB-α in LPS-stimulated N9 microglia cells. N9 microglial cells were pre-treated with 100 ng/ml NRG-1 for 24 h followed by the absence or presence of LPS (10 μg/ml) for indicated time points. **a**, **b** Cell lysates were taken and were assayed using ELISA. Results were expressed as the mean +/− SD. *Asterisk* denotes significant difference compared to cells treated with only LPS (*p* < 0.05)

**Fig. 5:**
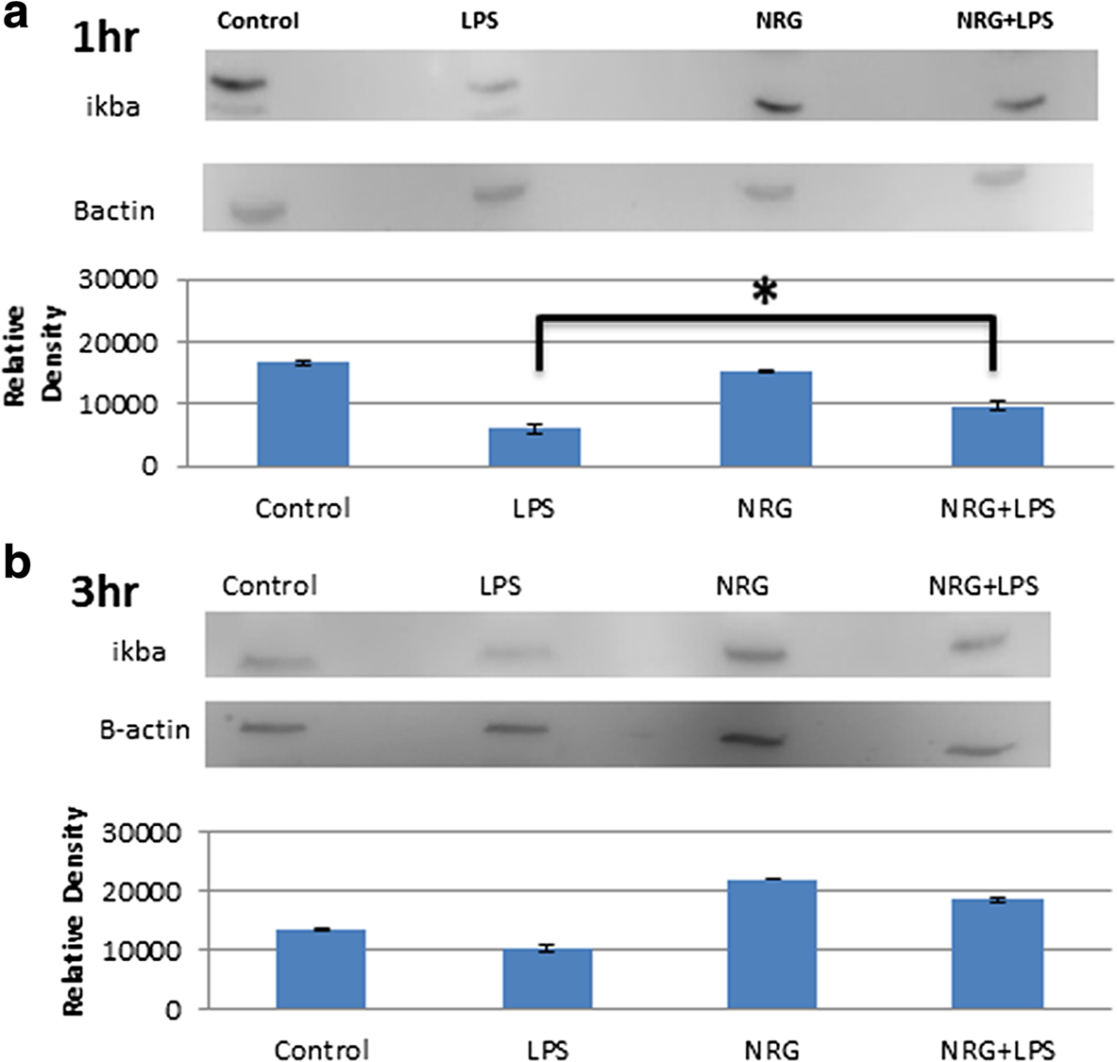
NRG-1 inhibits the degradation of IkB-α in LPS-stimulated N9 microglia cells. N9 microglial cells were pre-treated with 100 ng/ml NRG-1 for 24 h followed by the absence or presence of LPS (10 μg/ml) for 1 h (**a**) and 3 h (**b**). Whole cell extracts were taken from untreated cells or cells pre-treated NRG-1 alone or the absence or presence of LPS (10 μg/ml) and were assayed using western blot. The band intensity was quantified using studio lite imager and is presented relative to the level of β-actin. Data are presented for three independent experiments. Results were expressed as the mean +/− SD. *Asterisk* denotes significant difference compared to cells treated with only LPS (*p* < 0.05)

In the canonical NF-kB pathway, IkB kinase (IKK) phosphorylates IkB-α triggering its ubiquitination and degradation. This allows the NF-kB p56 and p50 subunits to translocate to the nucleus, resulting in the production and secretion pro-inflammatory cytokines and chemokines. Here, we examined whether NRG-1 regulates LPS-stimulated IkB-α phosphorylation in N9 microglia cells. N9 cells were either pre-treated with NRG-1 for 24 h followed by stimulation with LPS or treated with LPS or NRG-1 alone for the indicated times (1–24 h). Using an ELISA system, phosphorylation of IkB-α in microglial cell lysates increased 1 h post-LPS stimulation, peaked at 3 h, and then returned to baseline 6–24 h following LPS stimulation (Fig. 4a, b). Pre-treatment with NRG-1 significantly reduced the phosphorylation of IkB-α in response to LPS stimulation at both the 1- and 3-h time points. We used western blot analyses to determine the effect of NRG-1 on the degradation of IkB-α. N9 cells were pre-treated with NRG-1 for 24 h followed by stimulation with LPS or treated with LPS or NRG-1 alone for 1 and 3 h. At the l-h time point, there was a reduction in IkB-α in LPS-treated cells when compared to controls; however, this was reversed in cells pre-treated with NRG-1 followed by LPS treatment (*p* = 0.008) (Fig. 5).

#### NRG-1 attenuated P65 nuclear translocation in N9 microglia cells

**Fig. 6:**
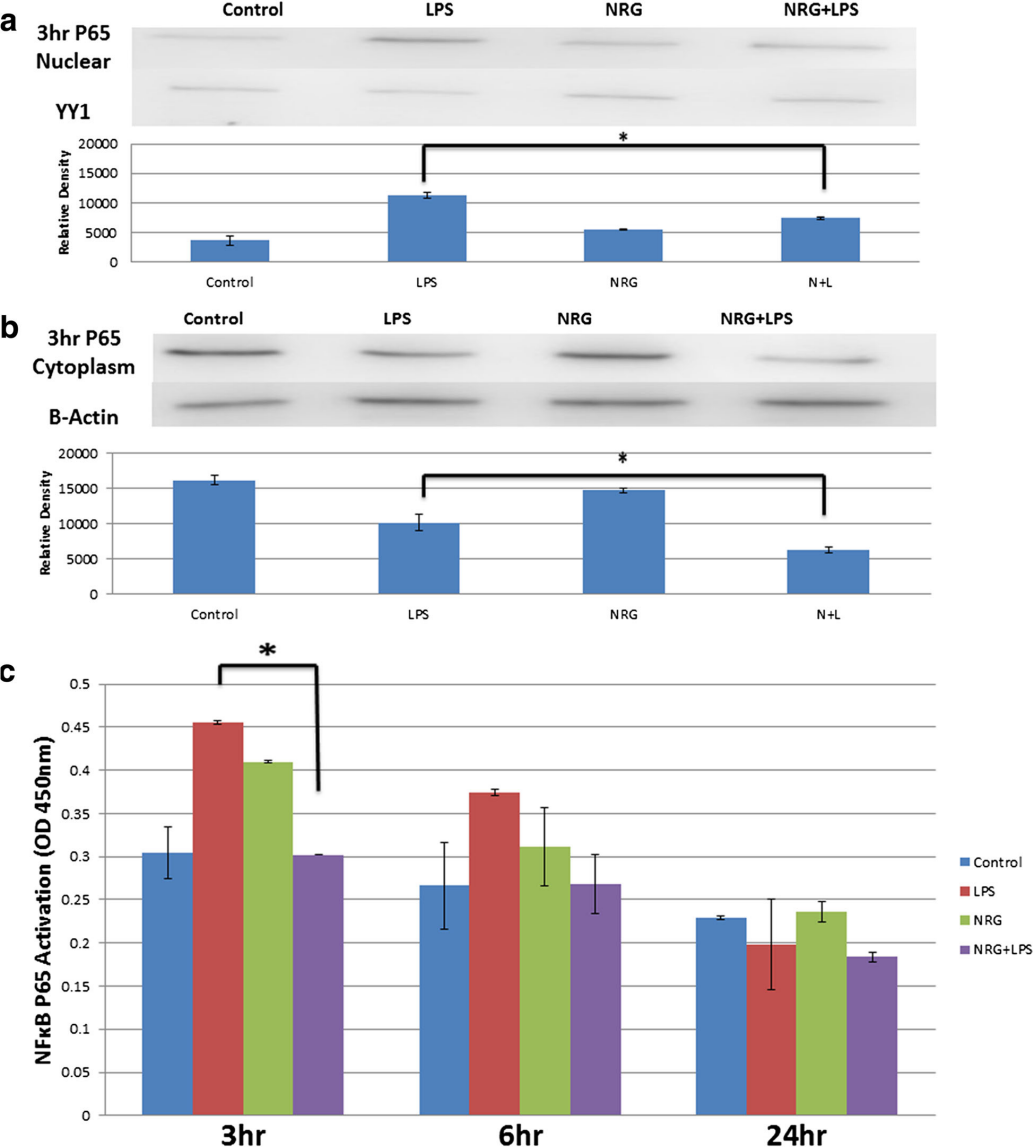
NRG-1 reduces nuclear translocation of P65. N9 microglial cells were pre-treated with 100 ng/ml NRG-1 for 24 h followed by the absence or presence of LPS (10 μg/ml) for 3 h. Nuclear (**a**) and cytoplasmic (**b**) extracts were assayed using western blot. Nuclear extracts were assayed using ELISA (**c**). Data are presented from three independent experiments. Results were expressed as the mean +/− SD. *Asterisk* denotes significant difference compared to cells treated with only LPS (*p* < 0.05)

To determine if NRG-1 had an effect on the canonical NF-kB pathway, we used nuclear extracts for ELISA and western blot to examine the translocation of the NF-kB p65 subunit identified by CONFAC. We observed an increase in nuclear translocation of p65 in N9 cells treated with LPS alone. However, LPS-induced p65 translocation was significantly reduced in cells pre-treated with NRG-1 followed by LPS using ELISA (Fig. 6a; *p* = 0.004) and western blot (Fig. 6b, *p* = 0.003).

#### NRG-1 increased the nuclear translocation of the P52 subunit

**Fig. 7:**
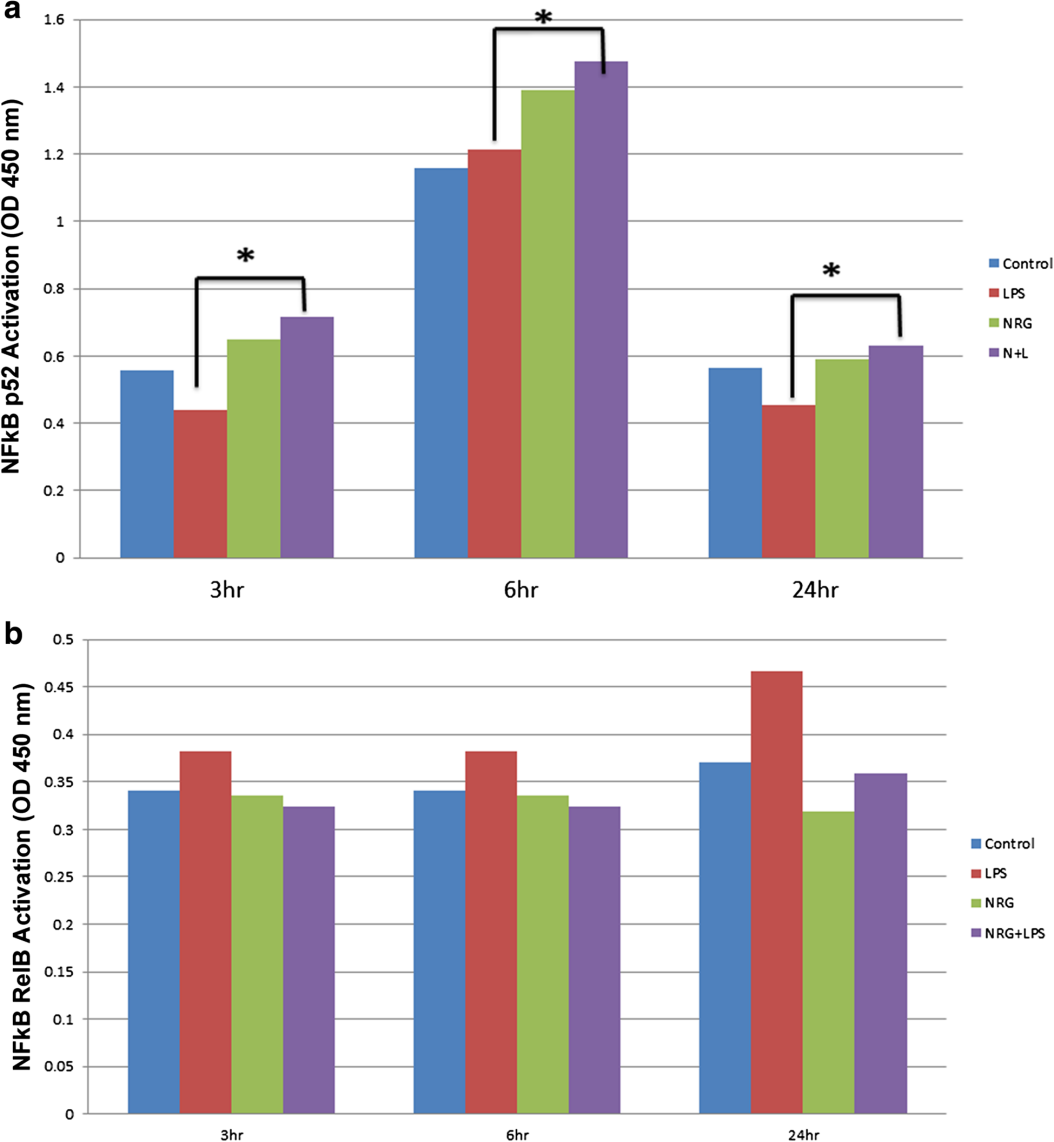
NRG-1 increased nuclear translocation of P52. N9 microglial cells were pre-treated with 100 ng/ml NRG-1 for 24 h followed by the absence or presence of LPS (10 μg/ml) for 3–24 h. Cell lysates were taken and were assayed using ELISA measuring levels of p52 (**a**) and RelB (**b**). Results were expressed as the mean +/− SD. *Asterisk* denotes significant difference compared to cells treated with only LPS (*p* < 0.05)

It has been previously shown that NRG-1 is able to activate NF-kB-inducing kinase (NIK) which is a mediator of the alternative NF-kB pathway [46]. Here, we investigated if NRG-1 could affect LPS-mediated nuclear translocation of the p52 and RelB NF-kB subunits. LPS stimulation resulted in a decrease in nuclear p52 at 3 and 24 h post-treatment (Fig. 7a). NRG-1 pre-treatment significantly increased nuclear p52 levels at 3, 6, and 24 h post-LPS stimulation. Neither LPS nor NRG treatment altered nuclear levels of RelB in these studies (Fig. 7b).

#### NRG-1 increased anti-apoptotic cytokines G-CSF and IL-9

**Fig. 8:**
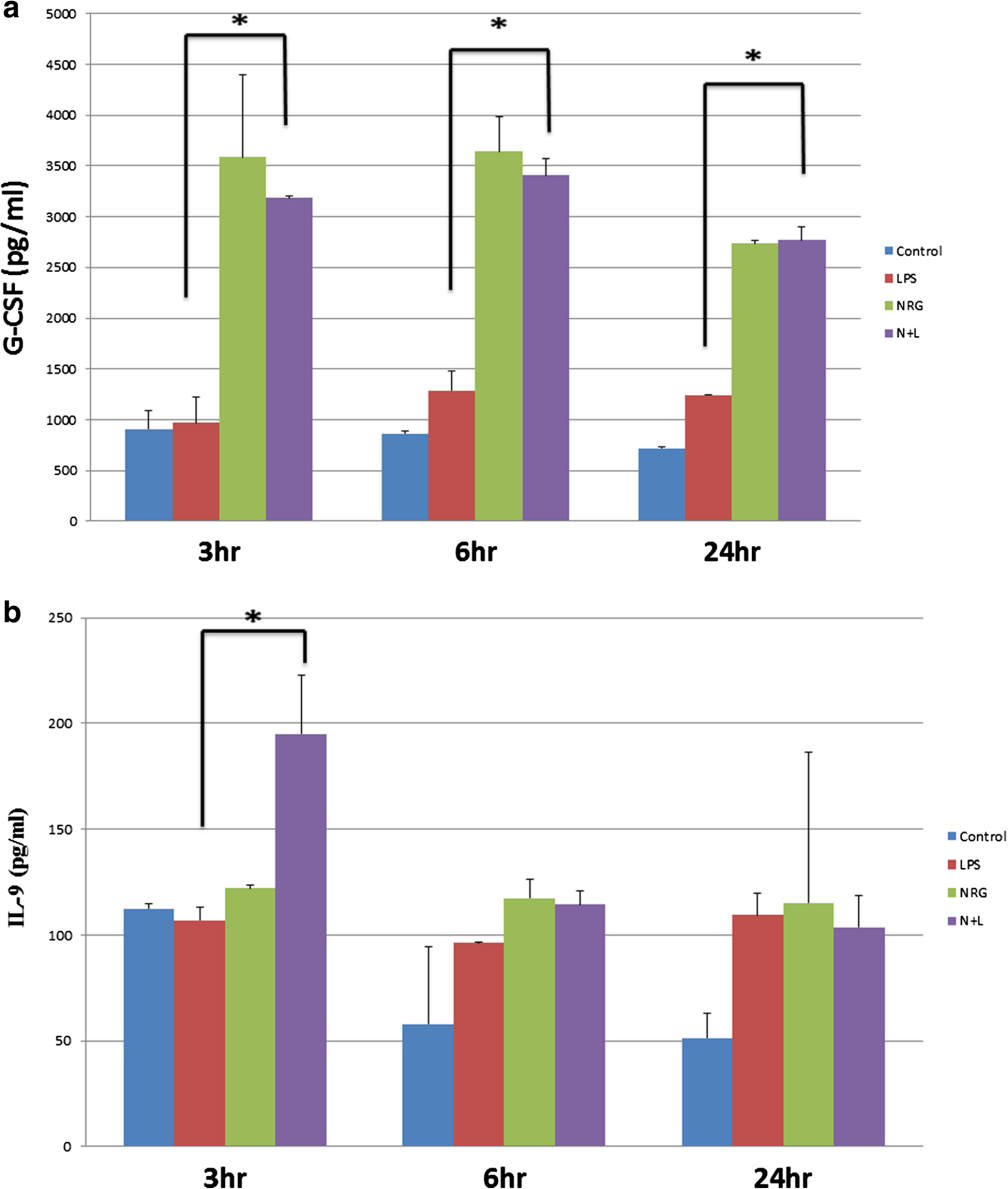
NRG-1 increases G-CSF and IL-9 concentrations in N9 microglia cells. N9 microglia cells were pre-treated with NRG-1 (100 ng/ml) for 24 h with or without LPS stimulation (10 μg/ml) for the indicated time points. Conditioned medium was collected and G-CSF (**a**) and IL-9 (**b**) levels were determined by Luminex. Results are expressed as the mean +/− SD. *Asterisk* denotes a significant difference compared to cells treated with only LPS (*p* < 0.05)

Granulocyte colony-stimulating factor (G-CSF) is a target of NF-kB and has been shown to be neuroprotective in stroke by inducing anti-apoptotic pathways [50–53]. The NF-kB inducible cytokine IL-9 has also been reported to prevent apoptosis. LPS stimulation of microglial cells did not affect G-CSF levels in medium. However, NRG-1 increased G-CSF levels significantly at 3, 6, and 24 h alone or in the presence of LPS (Fig. 8a). NRG-1 significantly increased IL-9 levels at 3 h following LPS stimulation (Fig. 8b). IL-9 was increased by all treatment conditions 6 and 24 h following NRG-1 and LPS administration.

## Discussion

NRG-1 has been shown to be neuroprotective and reduce inflammation in experimental models of ischemic stroke and neuroinflammation [1–3, 9, 10]; however, the molecular mechanism involved remains to be elucidated. Using CONFAC software, we were able to predict that the ability of NRG-1 to suppress ischemia-induced pro-inflammatory genes involved the modulation NF-kB pathways. NF-kB is known to be a master regulator of inflammation, which is the key contributor the pathology of ischemic stroke [24]. In post-mortem human brain samples of stroke patients, the NF-kB p65 subunit in particular has been found around the necrotic infarct core [22]. The NF-kB canonical pathway involves the phosphorylation and degradation of IkB leading to the nuclear translocation of p65/p50 NF-kB heterodimers to the nucleus where they stimulate the production of pro-inflammatory molecules [23, 24]. Using microglial cell cultures, we demonstrated that NRG-1 inhibited the LPS-induced phosphorylation and degradation of IkB-α, while also reducing the nuclear translocation of p65. This suggests that the mechanisms behind the anti-inflammatory/neuroprotective effects of NRG-1 involve the attenuation of the canonical NF-kB pathway.

The transcription factors identified by CONFAC did not change in mRNA expression in our analysis, including NF-kB family members. One of the primary reasons we chose a computational tool such as CONFAC is that it predicts transcription factors that change in *activity*, but not necessarily mRNA or protein levels. Indeed, induction of pro-inflammatory gene expression by NF-kB requires that cytoplasmic NF-kB p65/p50 translocates to the nucleus, but a change in NF-kB protein levels is not needed to mediate this effect. Over-representation of the NF-kB p65 TFBS indicates that it is more prevalent in the promoters of the genes in our dataset compared to a random set of genes. It does not indicate whether that promoter is activated or inhibited by the experimental paradigm. What our data suggest is that stroke causes transcriptional activation via the NF-kB p65-binding site, and NRG-1 prevents the binding of p65 to the TFBS by sequestering it in the cytoplasm.

The alternative NF-kB pathway is thought to have anti-inflammatory and anti-apoptotic roles [31, 35]. The alternative pathway activates NIK, a member of the MAP kinase family, which activates IKKα, resulting in the translocation of RelB/p52 NF-kB heterodimers to the nucleus. NIK-mediated activation of NF-kB is associated with the induction of anti-apoptotic and anti-inflammatory cytokines [30, 34, 37, 38, 50, 54, 55]. The alternative pathway is delayed relative to the canonical pathway, and only certain inducers are able to induce its activation [33]. In microglial cells, NRG-1 administration increased the nuclear levels of p52. In addition to dimerization with RelB, the p52 subunit is also able to homodimerize and considered to have a repressive role due to its lack of a transcription activation domain [32]. It has been previously shown that NRG-1 activates NIK [46]. These finding indicate that NRG-1 could both activate the alternative pathway and/or suppress NF-kB transcriptional activity via p52 [23, 32, 56].

Previous studies demonstrated that NRG-1 can activate the NF-kB signaling pathway cancer cells and Schwann cells [40–46]. In these studies, it appears that NRG-1 signals by activating NF-kB p65; however, the activation of NF-kB by NRG-1 in breast cancer cells is associated with the increased expression of anti-apoptotic genes. The discrepancy in the findings may be due to the differential expression of erbB receptors on the cells. In cancer cells and Schwann cells, NRG-1 signals through erbB2 and erbB3 receptors. Neuroprotection in stroke by NRG-1 is mediated by erbB4 which physically associates with NIK [46, 57]. Interestingly, NRG-1 alone has little effect on NF-kB in our in vitro studies. So, the anti-inflammatory effects of NRG-1 appear to be contextually related to the presence on a pro-inflammatory response.

Consistent with regulation of the alternative NF-kB pathway, NRG-1 induced the production of G-CSF and IL-9 in cell cultures. G-CSF has been shown to be neuroprotective in ischemic stroke models by inducing anti-apoptotic pathways, and IL-9 is known to promote cell proliferation and inhibit apoptosis [53, 58]. It has been reported that G-CSF significantly reduced the expression of microglial p65, reduced pro-inflammatory mediators, and promoted anti-inflammatory responses in models of multiple sclerosis [59, 60]. There are reports which suggest that IL-9 may have the ability to downregulate specific sets of genes induced by NF-kB by inducing the expression of BCL-3 [61]. BCL3 is an IkB protein that specifically associates with homodimers p50 and p52 and has been shown to regulate pro-survival genes. BCL3 is also able to repress transcription by increasing p52 homodimer binding to kB sites on DNA [62].

## Conclusions

Stroke is a leading cause of death and disability in the USA. There is an urgent need to better understand the pathophysiology associated with stroke in order to develop more effective therapies. Here, we show that the neuroprotective and anti-inflammatory effects of NRG-1 are associated with the regulation of both canonical and alternative NF-kB signaling pathways. NRG-1 is currently in clinical trials for heart failure and has been shown to be safe and efficacious in phase I and phase II patient studies [63, 64]. These findings have major implications for the development of NRG-1 as a clinical therapy for stroke and other inflammation-mediated disorders.

## Abbreviations

BSA: Bovine serum albumin
CONFAC: Conserved Transcription Factor-Binding Site Finder
DMEM: Dulbecco’s modified Eagle’s media
ECA: External carotid artery
ELISA: Enzyme-linked immunosorbent assay
FBS: Fetal bovine serum
ICA: Internal carotid artery
IL-1β: Interleukin-1β
IkB: Inhibitory kappa B
IKK: IkB kinase
IPA: Ingenuity Pathway Analysis
LPS: Lipopolysaccharide
MCA: Middle cerebral artery
MCAO: Middle cerebral artery occlusion
NF-kB: Nuclear factor kappa B
NIK: NF-kB-inducing kinase
NO: Nitric oxide
NRG-1: Neuregulin-1
PBS: Phosphate-buffered saline
PVDF: Polyvinylidene difluoride membranes
ROS: Reactive oxygen species
SDS-PAGE: Sodium dodecyl sulfate-polyacrylamide gel electrophoresis
TFBS: Transcription factor-binding sites
TNF-α: Tumor necrosis factor α
TTC: Triphenyltetrazolium chloride
